# SG-APSIC1126: Controlling SARS-CoV-2 infection in inpatients through a grouping system at Ho Chi Minh Children’s Hospital 1 in Vietnam

**DOI:** 10.1017/ash.2023.18

**Published:** 2023-03-16

**Authors:** Chau Nguyen Ngoc Minh, Thi Thanh Thuy Le, Thanh Hung Nguye, Ngoc Quang Minh Ngo, Van Niem Do, Thi Thanh Huong Nguyen

**Affiliations:** Children’s Hospital 1, Ho Chi Minh City, Vietnam; Ho Chi Minh Children’s Hospital 1, Ho Chi Minh City, Vietnam; Ho Chi Minh Children’s Hospital 1, Ho Chi Minh City, Vietnam; Ho Chi Minh Children’s Hospital 1, Ho Chi Minh City, Vietnam; Ho Chi Minh Children’s Hospital 1, Ho Chi Minh City, Vietnam; Ho Chi Minh Children’s Hospital 1, Ho Chi Minh City, Vietnam

## Abstract

**Objectives:** At the onset of COVID-19, whenever SARS-CoV-2 was detected at Children’s Hospital 1 (CH1), the related department or building was closed for extensive tracing, testing, and medical isolation. This process disrupted hospital activities, reduced the efficiency of patient care, and used medical resources. To address this problem, CH1 implemented a system of grouping inpatients to color-coded areas from June to December 2021. **Methods:** In this retrospective study, we describe the system of grouping inpatients to color-coded areas based on SARS-CoV-2 test result at a 1,600-bed, national pediatric hospital in Ho Chi Minh City. **Results:** Inpatients were first separated into those with or without respiratory symptoms, and secondly to different color-coded areas based on SARS-CoV-2 test result and hospitalization length: red zone (days 1–3), orange zone (days 3–7), and green zone (day 7 onward). Prior to admission, all patients were tested with a SARS-CoV-2 rapid diagnostic test. If negative, the patient was admitted to the red zone. On days 3 and 7 of hospitalization, the patient was tested using a pooled RT-PCR method. Patients negative on day 3 were relocated to the orange zone; patients negative on day 7 were relocated to the green zone. A patient with a positive test result at any time point was transferred to a COVID-19 zone. One caregiver was allowed to stay with 1 patient with similar testing regimen. A mobile transportation team was set up to deliver food and other necessities; thus, movement was restricted and interaction was prevented among zones. After this system was implemented, COVID-19 cases were detected early, with most positive cases in the red zone (19.6%) and the orange zone (2.8%), with only 1 case in the green zone (0.7%). **Conclusions:** The system of grouping patients to color-coded areas helped prevent SARS-CoV-2 transmission within the hospital, allowing undisrupted operation.

